# Long-Chain Fatty Acid Receptors Mediate Relaxation of the Porcine Lower Esophageal Sphincter

**DOI:** 10.3389/fphys.2019.00676

**Published:** 2019-05-31

**Authors:** Ching-Chung Tsai, Yi-Chen Li, Li-Ching Chang, Shu-Leei Tey, Kai-Jen Lin, Shih-Che Huang

**Affiliations:** ^1^Department of Pediatrics, E-Da Hospital, Kaohsiung City, Taiwan; ^2^School of Medicine for International Students, I-Shou University, Kaohsiung City, Taiwan; ^3^Department of Medical Research, E-Da Hospital, Kaohsiung City, Taiwan; ^4^Department of Pathology, E-Da Hospital, Kaohsiung City, Taiwan; ^5^Department of Internal Medicine, Shosanbetsu Village Clinic, Hokkaido, Japan

**Keywords:** motility, lower esophageal sphincter, free fatty acid receptor, FFA1, FFA4

## Abstract

Long-chain fatty acids activate the free fatty acid receptor 1 (FFA1) and FFA4. In the gastrointestinal system, FFA1 and FFA4 have been found in the pancreas and intestine. Fatty food and decreased lower esophageal sphincter (LES) motility are associated with gastroesophageal reflux disease. The effect of long-chain fatty acids on the esophageal motility is unknown. The purpose of this study is to investigate the effects of long-chain fatty acids on the porcine LES motility *ex vivo* using isometric transducers. In endothelin 1-precontracted porcine LES strips, the FFA1 selective agonists, fasiglifam, TUG424, and GW9508, caused marked relaxations in a concentration-dependent manner. The relative efficacies to elicit relaxation were GW9508 > TUG424 > fasiglifam in both clasp and sling strips. In contrast, the FFA4 specific agonists, TUG891 and GSK137647, produced mild relaxations. In addition, the endogenous FFA1 agonist DHA caused a mild relaxation whereas GW1100, an FFA1 antagonist, inhibited GW9508 induced relaxation of the porcine LES clasp and sling muscle. Both real-time PCR and immunohistochemistry revealed that FFA1 and FFA4 were expressed in the porcine LES. Real-time PCR analysis showed that the FFA4 expression was much lower than FFA1. Taken together, long-chain fatty acid receptor agonists elicit relaxation of the porcine LES. FFA1 might influence LES motility.

## Introduction

The prevalence of GERD has been reported to be increasing. Possible risk factors such as obesity, diabetes, smoking, alcohol, pregnancy, hiatal hernia, delayed stomach emptying, NSAIDs use, and sweet or fatty food are associated with GERD (Jarosz and Taraszewska, 2014; Asl et al., 2015). The sustained closure of LES can prevent esophagus from damage caused by gastric acid reflux. LES maintains tonic contraction modulated by myogenic, neural, and neurohumoral factors (Farré and Sifrim, 2008). The main underlying mechanism of GERD is LES incompetence that is caused by transient LES relaxations or decreased LES resting tone. In addition, some drugs, food or cigarette smoking may decrease LES pressure (Bredenoord et al., 2005; Farré, 2013). So far, the treatment of GERD has been focused on inhibition of acid secretion in the stomach, or acid neutralization. These medicines include antacids, histamine 2 receptor blockers, and proton pump inhibitors, but this approach fails to address LES incompetence that is one of the predominant mechanisms of GERD. Some patients with GERD are refractory to acid neutralization and secretion inhibition. Other medications to strengthen the LES, such as baclofen, are few and baclofen has neurological side effects that are a burden for clinical use (Yasawy and Randhawa, 2014).

The fatty food association with GERD may be related to a drop in LES pressure and slow gastric emptying caused by fatty food (Nebel and Castell, 1972, 1973). Free fatty acids can signal through interactions with G-protein coupled free fatty acid receptors. Long-chain fatty acids activate the free fatty acid receptor 1 (FFA1) and FFA4 (Hara et al., 2014; Milligan et al., 2015), whereas short-chain fatty acids stimulate FFA2 and FFA3 receptors. In the gastrointestinal system, these receptors are considered to have additional biologic functions (Regard et al., 2008; Bolognini et al., 2016). FFA2 and FFA3 receptors have been found in pancreatic β-cells and the intestine. Short-chain fatty acids have an effect on motility of the gastrointestinal tract, especially on the ileum (Richardson et al., 1991; Cherbut et al., 1997). FFA1 and FFA4 have been found in the pancreas and intestine (Itoh et al., 2003; Hirasawa, 2015). FFA1 is expressed mainly in the β-cells of the pancreas, the brain, and in a portion of the intestine. FFA1 regulates insulin secretion from β-cells of the pancreas. Activation of FFA1 also increases secretion of incretins from gut enteroendocrine cells. FFA4 is also a member of G protein-coupled receptors. It is expressed in the macrophage, colon, adipose tissue and tongue. FFA4 can promote the release of glucagon-like peptide-1 from the intestine, increase insulin sensitivity, and enhance glucose intake in adipocytes (Hirasawa, 2015; Milligan et al., 2015). Only a few studies regarding the effects of long-chain fatty acids on the gastrointestinal motility have been reported, and the effects of long-chain fatty acids on the LES motility have yet to be reported. Recently, potent and specific agonists for FFA1 and FFA4 have been developed as a potential medicine for diabetes (Watterson et al., 2014; Azevedo et al., 2016). Fatty food is associated with acid reflux in some patients with GERD (Jarosz and Taraszewska, 2014). In this study, we used these potent agonists of FFA1, fasiglifam, TUG424 and GW9508, and agonists of FFA4, TUG891 and GSK137647, to investigate the effects of long-chain fatty acids on the porcine LES motility. Furthermore, the potential about FFA1 antagonists in the treatment of GERD was discussed.

## Materials and Methods

All procedures were carried out in accordance with relevant laws in Taiwan and institutional guidelines of E-Da hospital. The pigs weighing approximately 110 kg were stunned and killed by exsanguination in a commercial slaughterhouse in accordance with guidelines of Council of Agriculture, Executive Yuan, R.O.C. (Taiwan). The pig stomach and lower esophagus was purchased from this slaughterhouse in Kaohsiung City, Taiwan. Because our purchased stomach and esophageal sphincter from a local slaughterhouse is food and not live animal, the study was exempt from review by the Institutional Animal Care and Use Committee (IACUC) of E-DA Hospital.

The proximal part of the stomach with the distal esophagus was cut and placed in ice-cold Krebs–Henseleit buffer solution that had the following composition: 4.7 mM KCl, 1.2 mM NaH_2_PO_4_, 25 mM NaHCO_3_, 118 mM NaCl, 1.8 mM CaCl_2_, and 14 mM glucose, pH 7.4, and transported on ice to the laboratory. The stomach collecting and processing time, from slaughtering, was approximately 30 min and transportation time was approximately 30 min. TUG891, GW9508, and fasiglifam (TAK875) were purchased from Cayman Chemical (MI, United States). TUG424 and GSK137647 were obtained from Tocris Bioscience (Bristol, United Kingdom). DHA was purchased from Abcam (MA, United States). GW1100 was obtained from Sigma-Aldrich (MO, United States). Anti-human FFA1 (GTX100290, lot # 39428) and FFA4 (GTX100364, lot # 41731) rabbit polyclonal antibodies were purchased from GeneTex Inc. (CA, United States). Bond Dewax Solution, Bond Epitope Retrieval Solution 1, Bond peroxide block, Bond polymer, Bond DAB (3,3′-diaminobenzidine tetrahydrochloride) and Bond hematoxylin were all purchased from Leica Biosystems Newcastle Ltd. (Newcastle-Upon-Tyne, United Kingdom).

### Measurement of FFA1 and FFA4 Agonists on Relaxation of the LES Sling and Clasp Muscles in Pig

The porcine LES, which has similar structure to the human LES, was divided into clasp with LC and sling with GC muscle strips according to previously published procedures with minor modifications (Farré et al., 2007; Tsai et al., 2015, 2018). The porcine LES is often used as an animal model system to study esophageal motility and GERD in humans (Korn et al., 1997; Schopf et al., 1997). The fresh porcine stomach was cut longitudinally along the midline between the GC and LC of the stomach. The mucosa was removed. Lower esophageal sling and clasp muscles were obtained in accordance with the previously reported method (Farré et al., 2007). The muscle strips (each 3 mm wide and 10 mm long) were pruned from the area of the sling and clasp muscles separately. Subsequently, the muscle strips were mounted in 7 ml organ baths holding 5 ml of Krebs solution at 37°C and continuously bubbled with 95% O_2_ + 5% CO_2_. The muscle strips were connected with surgical silk sutures to isometric transducers (FORT10g; Grass Technologies, RI, United States), which were connected to an amplifier (Gould Instrument Systems, OH, United States). Then, the signal was recorded by a computer recording system (BIOPAC Systems, CA, United States). The basal tone of the muscle strip was adjusted to be 1.0 g. After a 30 min equilibration period, 1 × 10^−6^ M carbachol was added into the organ bath to check the contraction of the muscle strips, and then, the carbachol was washed out. When reaching another equilibration period, endothelin 1 (3 × 10^−7^ M), which induced a long-duration muscle contraction (Tsai et al., 2018), was added to induce a contraction. Sequentially 1 × 10^−5^, 3 × 10^−5^, 1 × 10^−4^, and 3 × 10^−4^ M GW9508, fasiglifam, TUG424, TUG891, or GSK137647 was added in a non-cumulative dose manner into the organ baths separately, and the relaxation responses of isolated sling or clasp strips were recorded. Each LES strip from the same animal was used for a different dose of agonist. Furthermore, 1 × 10^−4^ M and 1 × 10^−3^ M endogenous agonist DHA was added in a non-cumulative dose manner in endothelin 1-precontracted porcine LES sling and clasp fibers, respectively. Methanol was used as the solvent for GW9508, fasiglifam, TUG424, TUG891, GSK137647, and DHA.

### Inhibition of GW9508-Induced Relaxation of Porcine LES Sling and Clasp Strips by a Selective FFA1 Antagonist, GW1100

To investigate whether FFA1 is involved in long-chain fatty acid-induced relaxation of LES, the endothelin 1-precontracted sling and clasp strips were treated with a selective FFA1 antagonist GW1100 (1 × 10^−5^ M), and 15 min later, GW9508 (1 × 10^−4^ M) was added into the organ baths and the relaxation responses of isolated muscle strips were recorded (Moodaley et al., 2017).

### Real-Time Polymerase Chain Reaction (PCR) Analysis of FFA1 and FFA4 in the LES

Sling and clasp muscles were trimmed from the LES, stored in a solution of RNAlater^®^ (Applied Biosystems Inc., Foster City, CA, United States) at 4°C for 2 days for tissue penetration. Then, the excess liquid was removed, and the tissue was preserved at −80°C.

RNA was extracted from tissue samples by the guanidine isothiocyanate process using the GeneJET RNA Purification Kit (Thermo Fisher Scientific Inc., Vilnius, Lithuania) in accordance with the manufacturer’s protocol. A spectrophotometer (DU800^®^ UV–Vis, Beckman Coulter, CA, United States) was used to monitor the quality and purity of the isolated RNA.

After measurement of the RNA concentration, the cDNA was synthesized using the isolated RNA and the High Capacity cDNA Reverse Transcription Kit for RT-PCR (Applied Biosystems Inc., CA, United States). The synthesis of cDNA from an RNA template via reverse transcription was performed on ice in accordance with the manufacturer’s protocol. A volume corresponding to 2 μg of RNA was used for subsequent synthesis. The samples containing cDNA were diluted with DNase/RNase-Free distilled water in a volume ratio of 1:9 and prepared for subsequent real-time PCR.

The real-time PCR amplification reactions were carried out with an Applied Biosystems^®^ 7500 Real-Time PCR System (Applied Biosystems, MA, United States) using the QuantiFast SYBR Green PCR Master Mix (Thermo Fisher Scientific Inc, MA, United States) under the following programming: one cycle of 95°C for 5 min, 40 cycles of 95°C for 10 s, and 60°C for 45 s. The primer sequences were: (1) FFA1: forward primer – TGGGAGGCTACTGGCGGAAG and reverse primer – GCCACACATGTTGTCCCCCG; (2) FFA4: forward primer – GTGCTGGCTGTTCGCTGGAC and reverse primer – AAGGCGCACAATGCACACCA. The 2^−Δ^ ^Δ^ ^Ct^ method was used to analyze the relative changes in gene expression from real-time quantitative PCR studies (Chiang et al., 2010; Grassin-Delyle et al., 2013; Tsai et al., 2018).

### Immunohistochemistry (IHC)

Immunostaining with anti-FFA1 (polyclonal, GeneTex, CA, United States) and anti-FFA4 (polyclonal, GeneTex, CA, United States) antibodies were executed on the fully automated Bond-Max system (Leica Microsystems, Nussloch, Germany) according to previously described procedures with minor modifications (Tsai et al., 2015, 2018). Slides carrying tissue sections of porcine sling and clasp muscles, cut from paraffin-embedded, formalin-fixed sections, were dried for 30 min at 60°C. These slides were then covered by Bond Universal Covertiles and placed into the Bond-Max instrument. All subsequent steps were performed by the automated instrument according to the manufacturer’s instructions (Leica Microsystems), in the following procedures: (1) deparaffinization of tissue on the slides by rinsing with the Bond Dewax Solution at 72°C; (2) heat-induced epitope retrieval (antigen unmasking) with the Bond Epitope Retrieval Solution 1 for 20 min at 100°C; (3) peroxide block placement for 5 min at room temperature; (4) incubation with rabbit polyclonal anti-FFA1 antibody at a dilution of 1:100 or anti-FFA4 antibody at a dilution of 1:500 for 30 min at room temperature; (5) Bond Polymer placement for 8 min at room temperature; (6) color development with DAB as a chromogen for 5 min at room temperature; and (7) haematoxylin counterstaining for 5 min, followed by mounting of the slides and examination by light microscopy. The negative control sections were stained with normal rabbit IgG at equimolar concentrations.

### Data Analysis

The data were expressed as the means ± SEM. Statistical analysis of the results was performed by using one-way ANOVA, followed by Dunnett’s procedure. In all cases, differences were considered significant when *p* < 0.05. GraphPad Prism 5 (GraphPad Software, Inc.) was used to determine the EC50 values.

## Results

### Selective FFA1 and FFA4 Agonists Cause Relaxation of the Porcine LES Sling and Clasp Muscles

**FIGURE 1:**
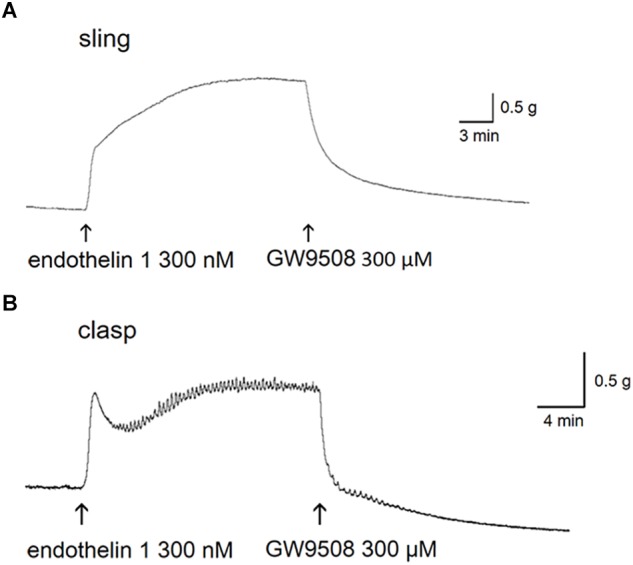
Relaxant tracings of GW9508 in endothelin 1-precontracted lower esophageal sphincter. Representative relaxant tracings of porcine lower esophageal sphincter sling **(A)** and clasp **(B)** muscle strips induced by GW9508. The arrows indicate the addition of endothelin 1 and GW9508.

In muscle strips isolated from the porcine LES, 3 × 10^−7^ M endothelin 1 induced a marked and long-duration muscle contraction. GW9508 was tested in sling and clasp muscles at rest or precontracted with endothelin 1. Although 3 × 10^−4^ M GW9508 caused mild relaxation in LES sling and clasp muscles at rest (24.0 ± 6.0 and 30.0 ± 6.3% of 1 × 10^−6^ M carbachol-induced tone, respectively; both *n* = 3), there was no significant difference compared with its vehicle (60% methanol)-induced relaxation (*p* > 0.05, 12.2 ± 1.6 and 18.0 ± 3.1% of 1 × 10^−6^ M carbachol-induced tone, respectively; *n* = 3 and 6). In contrast, in sling and clasp muscles, 3 × 10^−4^ M GW9508 induced an obvious muscle relaxation of the endothelin 1-precontracted porcine LES sling and clasp strips (Figure 1). In addition, in endothelin 1-precontracted porcine LES sling and clasp strips, the FFA1 selective agonists, fasiglifam and TUG424, also caused similar marked relaxations.

**FIGURE 2:**
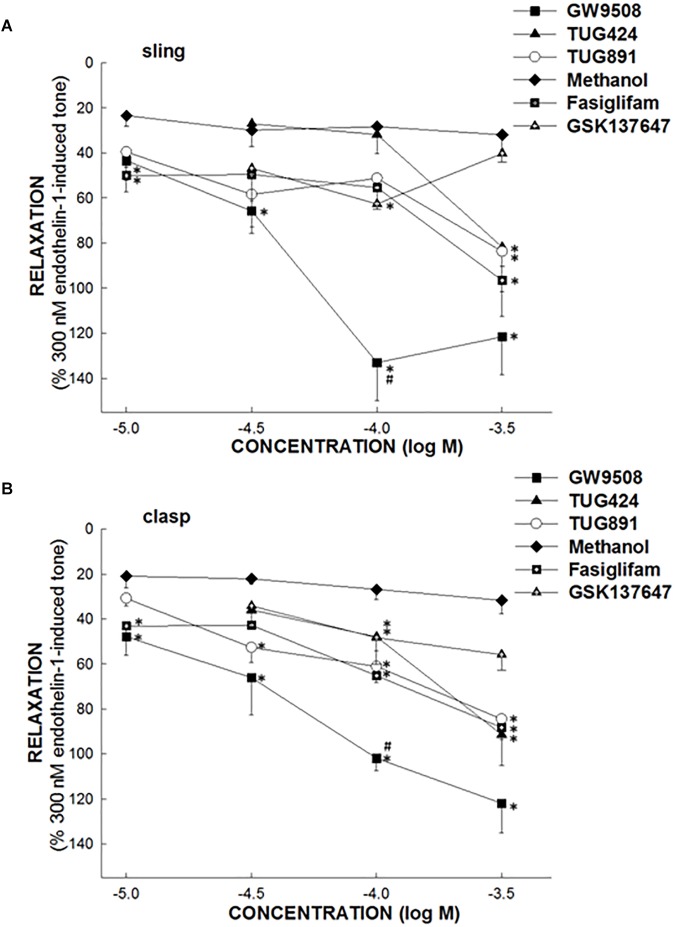
The effect of FFA1 and FFA4 agonists on porcine lower esophageal sphincter. The ability of specific free fatty acid receptor 1 (FFA1) agonists, FFA4 agonists or methanol (vehicle) to induce relaxation of the porcine lower esophageal sphincter (LES) sling **(A)** and clasp **(B)** muscle strips. The FFA1 specific agonists, GW9508, TUG424, and fasiglifam induced marked relaxation of the porcine LES muscle in a dose-dependent manner. The FFA4 specific agonists, TUG891 and GSK137647, produced mild relaxations of the porcine LES muscle. The values are expressed as a percent of an endothelin 1 (300 nM)-induced contraction. The results given are from at least four experiments. The vertical bars represent ± standard error of the mean (SEM). The ^∗^ represents a significant difference compared with the relaxation induced by an equivalent concentration of methanol (*p* < 0.05). The # indicates a significant difference compared with the relaxation induced by an equivalent concentration of fasiglifam (*p* < 0.05).

In endothelin 1-precontracted porcine LES sling and clasp strips, 1 × 10^−5^, 3 × 10^−5^, 1 × 10^−4^, and 3 × 10^−4^ M GW9508, fasiglifam, TUG424, TUG891, and GSK137647, respectively, caused relaxations in a concentration-dependent manner. The relative efficacies of FFA1 selective agonists to elicit relaxation were GW9508 > fasiglifam ≥ TUG424 in both sling and clasp strips. The EC50 of GW9508 causing maximal relaxation was 3.53 × 10^−5^ M in sling strips. However, the FFA4 specific agonists, TUG891 and GSK137647, produced mild relaxations, and the relative efficacy to cause relaxation was TUG891 > GSK137647 in both clasp and sling strips (Figure 2; *n* ≥ 4). This suggests that FFA1 and FFA4 agonists can cause relaxation of the porcine LES.

In endothelin 1-precontracted porcine LES, 1 × 10^−4^ M of the endogenous agonist DHA did not induce significant relaxation compared with vehicle, 100% methanol. However, 1 × 10^−3^ M DHA caused a significant relaxation, 73.3 ± 5.2 and 78.1 ± 6.9% of 3 × 10^−7^ M endothelin 1 induced tone in sling and clasp strips (*n* = 7 and 4), respectively [*p* < 0.05, compared with 100% methanol alone, 15.9 ± 2.9 and 17.2 ± 7.4% in sling and clasp strips (*n* = 6 and 4), respectively].

### Inhibition of GW9508-Induced Relaxation of the Porcine LES Sling and Clasp by a Selective FFA1 Antagonist, GW1100

**FIGURE 3:**
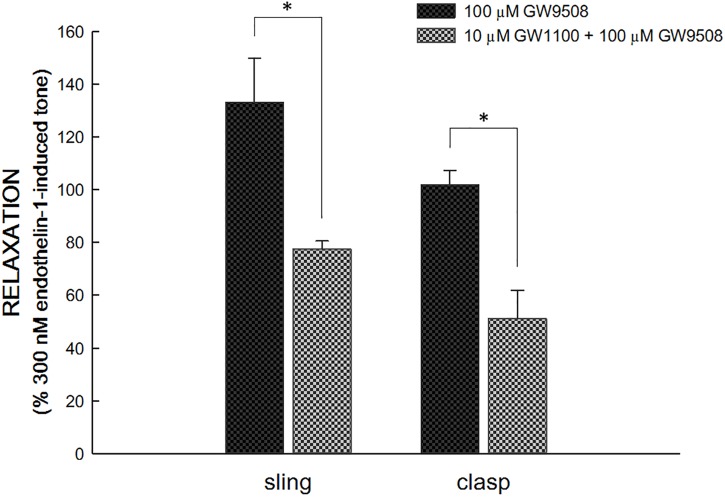
Inhibition of GW1100, a selective FFA1 antagonist, on GW9508-induced relaxation in the porcine LES sling and clasp muscles. GW1100 (1 × 10^−5^ M), the selective FFA1 antagonist, inhibited the relaxation caused by GW9508 (1 × 10^−4^ M) in endothelin 1-precontracted porcine LES sling and clasp muscles. With GW1100, GW9508 generated a significantly smaller relaxation. The ^∗^ represents a significant difference compared with the relaxation induced by GW9508 alone (*p* < 0.05).

In endothelin 1-precontracted porcine LES, 1 × 10^−5^ M GW1100, the selective FFA1 antagonist, inhibited the relaxation caused by 1 × 10^−4^ M GW9508. Specifically, with GW1100, GW9508 generated a significantly smaller relaxation, 77.5 ± 3.1 and 51.1 ± 10.8% of 3 × 10^−7^ M endothelin 1 induced tone in sling and clasp fibers (*n* = 6 and 4), respectively [*p* < 0.05, compared with GW9508 alone, 133.1 ± 16.7 and 101.9 ± 5.4% in sling and clasp fibers (both *n* = 6), respectively; Figure 3]. At rest, 1 × 10^−5^ M GW1100 didn’t cause relaxation or contraction of the LES sling and clasp muscles (data not shown).

### Real-Time PCR

**FIGURE 4:**
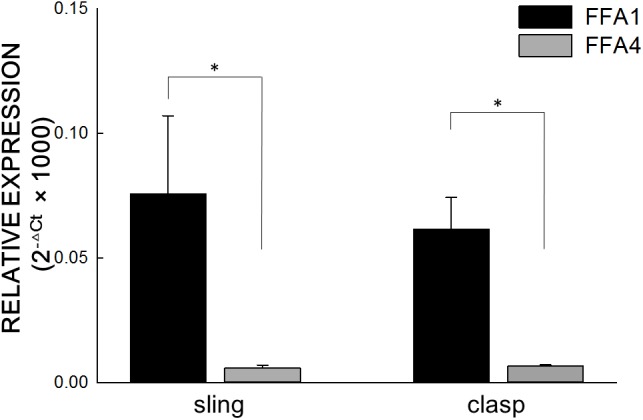
Real-time polymerase chain reaction of FFA1 and FFA4. Real-time polymerase chain reaction analysis revealed that free fatty acid receptor 1 (FFA1) and FFA4 were expressed in the clasp and sling muscles of the porcine lower esophageal sphincter. The relative FFA expression levels in the porcine lower esophageal sphincter were calculated using the comparative CT method and normalized against two housekeeping genes, glyceraldehyde 3-phosphate dehydrogenase and β-actin. Vertical bars represent ± standard error of the mean (*n* = 6/group). Compared to FFA1, the FFA4 expression is significantly much lower in the clasp and sling muscles (both *n* = 6). The ^∗^ represents a significant difference compared with FFA1 (*p* < 0.05).

Real-time PCR analysis revealed that FFA1 and FFA4 were expressed in the clasp and sling muscle of the porcine LES (Figure 4). Compared to FFA1, the FFA4 expression is significantly much lower in the clasp and sling muscles (both *p* < 0.05 and *n* = 6).

### Immunohistochemistry (IHC)

**FIGURE 5:**
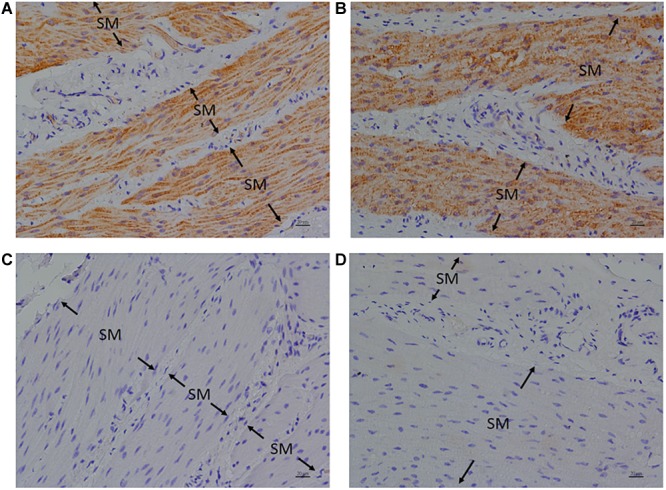
Immunohistochemical studies with an anti-FFA1 antibody on porcine lower esophageal sphincter. Tissue sections were stained with an anti-FFA1 (GTX100290) antibody and stained with DAB (3,3′-diaminobenzidine tetrahydrochloride) as a chromogen. Free fatty acid receptor 1 immunostaining was detected in the smooth muscle (SM) of the paraffin-embedded LES sling **(A)** and clasp **(B)** muscles (magnification 400×). The negative controls in the sling **(C)** and clasp **(D)** muscle were stained with equimolar concentrations of normal rabbit IgG (magnification 400×).

**FIGURE 6:**
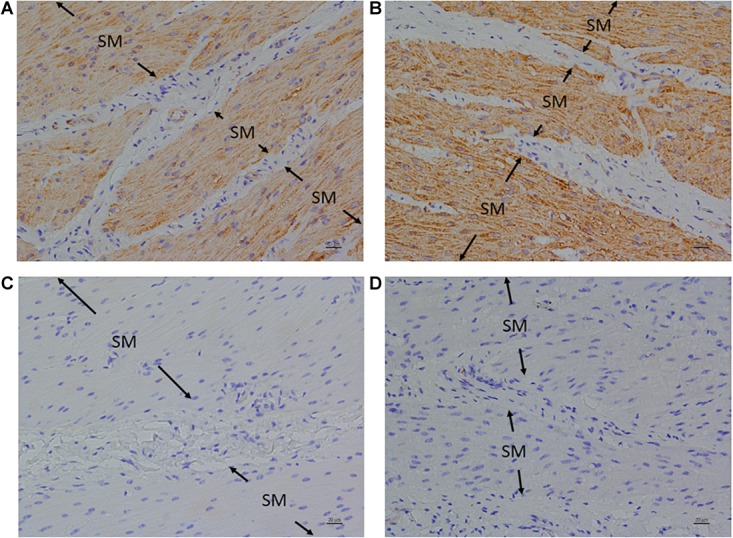
Immunohistochemical studies with an anti-FFA4 antibody on porcine lower esophageal sphincter. Tissue sections were incubated with an anti-FFA4 (GTX100364) antibody and stained with DAB (3,3′-diaminobenzidine tetrahydrochloride) as a chromogen. Free fatty acid receptor 4 immunostaining was detected in the smooth muscle (SM) of the paraffin-embedded LES sling **(A)** and clasp **(B)** muscles (magnification 400×). The negative controls in the sling **(C)** and clasp **(D)** muscle were stained with equimolar concentrations of normal rabbit IgG (magnification 400×).

Anti-FFA1 and anti-FFA4 antibodies were used to detect FFA1 and FFA4 separately in the porcine LES. IHC results revealed that the FFA1 was ubiquitous in the LES sling (Figure 5A) and clasp (Figure 5B) muscle fibers (*n* = 3). In contrast, no staining was detected in the negative controls, which were stained with equimolar concentrations of normal rabbit IgG in the sling (Figure 5C) and clasp (Figure 5D) muscle. In addition, IHC results also revealed that the FFA4 was ubiquitous in the LES sling (Figure 6A) and clasp (Figure 6B) muscle fibers (*n* = 3). Conversely, no staining was detected in the negative controls of the sling (Figure 6C) and clasp (Figure 6D) muscle.

## Discussion

Free fatty acid receptor 1 and FFA4 are G-protein coupled receptors activated by long-chain free fatty acids. FFA1 and FFA4 have been detected in the gastrointestinal tract and pancreas (Itoh et al., 2003; Hirasawa, 2015; Ren et al., 2016). FFA1 induces secretion of cholecystokinin in the duodenum in response to dietary fat (Liou et al., 2011). FFA1 and FFA4 are also found in the taste buds, and mediate the taste of fatty acids (Cartoni et al., 2010). Few studies about long-chain fatty acids affecting intestinal motility have been reported. The long-chain (n-3) polyunsaturated fatty acids (PUFA) may regulate the contractility of the small intestine (Patten et al., 2002). Long and medium-chain free fatty acids induce an increase in plasma cholecystokinin concentrations, modify gastric motility (McLaughlin et al., 1999) and inhibit jejunal motility. To our knowledge, studies regarding long-chain fatty acid on LES motility have not been reported.

In this study, we found that specific FFA1 and FFA4 agonists can induce relaxation of the porcine LES, and the relaxation is concentration-dependent. Specific FFA1 agonists, especially GW9508, can cause marked relaxation of the porcine LES sling and clasp muscle fibers. In addition, the endogenous FFA1 agonist DHA caused a relaxation whereas GW1100, an FFA1 antagonist, inhibited GW9508-induced relaxation of the porcine LES sling and clasp muscles. Furthermore, real-time quantitative PCR and IHC showed that FFA1 and FFA4 were expressed in the porcine LES. Compared to FFA1, the FFA4 expression is much lower. It is not clear whether the fatty acid receptor expressions could change in the LES depending on the time of the day or food intake. In this study, the FFA4 expression is too low to draw any conclusion on the effect of FFA4 agonists on the LES muscle relaxation. Taken together, these results suggest that the FFA1 might play an important part in mediating relaxation of the porcine LES sling and clasp muscle fibers. Further research is warranted to verify the relationship between long-chain fatty acid receptors and hypotensive LES pressure induced by fatty food.

Changes of cell functions by increased lipid intake might be related to the development of diabetes and cancer. Recently, FFA1 and FFA4 are considered to play an important role in diabetes and cancer (Houthuijzen, 2016). FFA1 and FFA4 are potential targets for treatment of type 2 diabetes (Watterson et al., 2014; Azevedo et al., 2016). In addition, fatty food might be associated with GERD. Possibilities include changes in gastrointestinal motility and/or gastric acid secretion (Nebel and Castell, 1972, 1973). In this study we found that fatty acids may induce LES relaxation. Our study indicates the potential of FFA1 antagonists in the treatment of GERD.

## Conclusion

In conclusion, long-chain fatty acid receptor agonists elicit relaxation of the porcine LES. FFA1 influences the LES relaxation. Long-chain fatty acid receptors might play an important role in the control of LES motility.

## Author Contributions

S-CH designed the study. C-CT, Y-CL, L-CC, and S-LT executed the study. C-CT, Y-CL, L-CC, S-LT, and S-CH analyzed and interpreted the data. C-CT and K-JL performed the immunohistochemistry. C-CT and S-CH wrote and edited the manuscript. All authors read the manuscript and given the final approval of the version to be published.

## Conflict of Interest Statement

The authors declare that the research was conducted in the absence of any commercial or financial relationships that could be construed as a potential conflict of interest.

## Abbreviations

DHA: docosahexaenoic acid
EC50: half-maximal contraction
FFA1: 2, 3, 4, free fatty acid receptor 1, 2, 3, 4
GC: greater curvature
GERD: gastroesophageal reflux disease
IHC: immunohistochemistry
LC: lesser curvature
LES: lower esophageal sphincter
PCR: polymerase chain reaction
PUFA: polyunsaturated fatty acids.
